# A Scalable Framework for Map Matching Based Cooperative Localization

**DOI:** 10.3390/s21196400

**Published:** 2021-09-25

**Authors:** Chizhao Yang, Jared Strader, Yu Gu

**Affiliations:** Department of Mechanical and Aerospace Engineering, West Virginia University, Morgantown, WV 26506, USA; jared.l.strader@gmail.com (J.S.); yu.gu@mail.wvu.edu (Y.G.)

**Keywords:** multi-robot systems, localization, sensor fusion, cooperating robots

## Abstract

Localization based on scalar field map matching (e.g., using gravity anomaly, magnetic anomaly, topographics, or olfaction maps) is a potential solution for navigating in Global Navigation Satellite System (GNSS)-denied environments. In this paper, a scalable framework is presented for cooperatively localizing a group of agents based on map matching given a prior map modeling the scalar field. In order to satisfy the communication constraints, each agent in the group is assigned to different subgroups. A locally centralized cooperative localization method is performed in each subgroup to estimate the poses and covariances of all agents inside the subgroup. Each agent in the group, at the same time, could belong to multiple subgroups, which means multiple pose and covariance estimates from different subgroups exist for each agent. The improved pose estimate for each agent at each time step is then solved through an information fusion algorithm. The proposed algorithm is evaluated with two different types of scalar field based simulations. The simulation results show that the proposed algorithm is able to deal with large group sizes (e.g., 128 agents), achieve 10-m level localization performance with 180 km traveling distance, while under restrictive communication constraints.

## 1. Introduction

Cooperative multi-agent systems have become increasingly popular due to the wide range of applications that they support, such as surveillance [1], search and rescue [2], and exploration [3]. In these applications, high-quality localization, the ability for agents to reliably and accurately estimate their poses (i.e., positions and orientations) with respect to the surrounding environment or to a geographic coordinate system, is crucial. The GNSS, as a traditional solution, is not always available or reliable, due to reasons such as signal blockages, multipath reflection, and jamming [4]. One potential solution for localization in GNSS-denied environments is to utilize map matching techniques, given a prior map represented as a scalar field. Scalar fields associate a scalar value with every point in space, and applications include gravity anomaly [5], magnetic anomaly [6,7], topographic [8], and olfaction [9], to name a few. Methods utilizing scalar fields for localization regulate agents’ dead-reckoning error growth through matching the information measured by on-board sensors with the prior given scalar field maps, such as terrain-aid navigation [10] and magnetic anomaly–based navigation [7]. However, these methods are sensitive to the characteristic information available in the local area near the agent, sensor noises, and the resolution and accuracy of the given maps. For example, for a single agent localization, the on-board sensor measurements could match to multiple positions on a scalar field map, creating ambiguity. This can be alleviated through matching the past sensor measurements along the agent’s trajectory to the map [11] but is still often not robust in real-world applications. Fortunately, compared to a single agent, a group of collaborative agents may provide several navigational benefits, such as tolerance against individual sensor failures. This can be achieved through sharing observations across a large spatial area on the scalar field. Therefore, cooperative localization using scalar field is an active research topic that has been studied over the last decade [12,13,14,15,16].

The scalar field–based cooperative localization algorithms can be classified into two main approaches. The first one is to treat the multi-agent group as a unity and to match observations from all agents with the given map to estimate their poses at each time step, which can be considered centralized methods. The centralized methods are able to achieve error growth bounded positioning and show robustness to issues such as low resolution of the map [6]. However, due to constraints on communication and the on-board computing resources for the agents, the group size of the centralized cooperative localization is limited in practice. The second approach is to perform decentralized cooperative localization, which means that each agent in the group estimates its own pose based on scalar field observations independently at each time step. Then, the estimates are updated, using the relative information (such as ranging, bearing) between this agent and its neighbors [12]. Usually, the communication constraints (e.g., range, connectivity, bandwidth) are considered when designing the decentralized localization approaches. In theory, decentralized methods are scalable to the group size and robust to errors made by, or failures of, individual agents. However, in the existing approaches [12,17], each individual agent needs to come up with a pose estimation, using its own scalar field measurement first, which has potential robustness issues in information poor regions. Thus, to develop a scalable cooperative localization algorithm that is robust to map matching errors while respecting practical communication constraints is the focus of this work.

**Figure 1:**
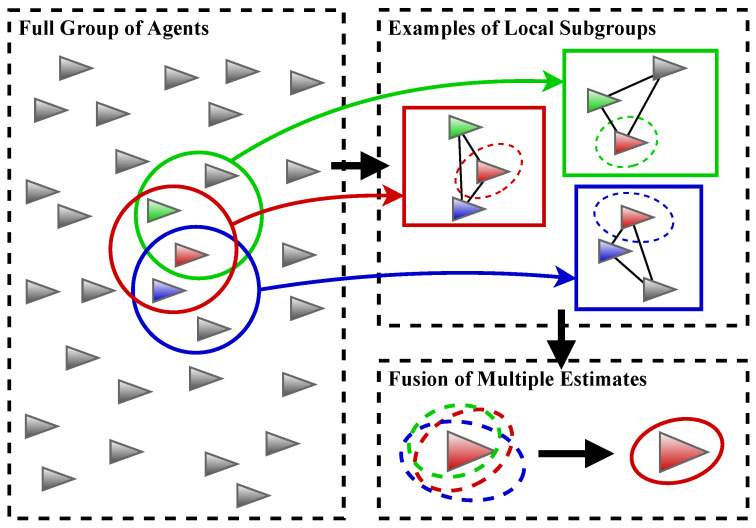
Illustration of the proposed framework for map matching based cooperative localization. (Left) A large group of agents is divided into subgroups based on communication constraints where one subgroup is created for each agent. (Upper right) The geometry of the subgroups (i.e., the relative positions inside the subgroups) are estimated, using range-only measurements, then the geometry is used to extract measurements of the scalar field to estimate the pose and associated uncertainty of each member in the subgroup. (Lower right) An agent, as an example, receives multiple copies of its pose estimate through its membership in several subgroups and fuses them to reduce pose error.

In this paper, a scalable framework is presented to perform cooperative localization based on scalar field information, which is performed through fusing the solutions estimated by smaller local subgroups in a large group. The general concept of the proposed approach is illustrated in Figure 1. The overall group is first organized into several subgroups. A subgroup is defined with one agent as the fusion center and a limited number of agents that the fusion center can communicate with as members, constrained by either the communication range or the number of available communication channels. In this case, the number of subgroups is the same as the number of agents in the group, and each agent belongs to several different subgroups at the same time. The inter-agent ranging measurements are assumed to be available within the subgroups. A locally centralized cooperative localization method is performed for each subgroup at the fusion center agent to estimate all members’ global pose and error covariance. Since each agent in the group belongs to multiple subgroups (as the fusion center or a member) at the same time, it can receive multiple global pose estimates and the corresponding covariances from fusion centers of these subgroups through the communication links. Eventually, each agent would gain an improved global pose estimate through applying a covariance intersection (CI) method to fuse these redundant estimates, using information provided by other subgroups.

The contributions of this paper are summarized as follows. First, the proposed algorithm can be scaled to large group sizes under communication constraints (e.g., a group of 128 agents was simulated) with a limitation that the cooperative localization performance is a function of the subgroup size instead of the full group size. Second, the simulation results demonstrate that the proposed algorithm can provide good localization performance for two different types of scalar fields based applications (i.e., magnetic anomaly matching for aerial vehicles and terrain matching for underwater vehicles). Third, compared with our previous works in [6,15], the proposed algorithm is shown to have improved performance with a similar computation cost for each agent in the group. Finally, the source code of the proposed algorithm and simulator is shared online to allow the readers to more easily verify and build on our work. The code is available online: https://github.com/wvu-irl/Scalable-Framework-Cooperative-Localization.git (Date accessed: 21 September 2021).

The rest of this paper is organized in the following manner. The related works are presented in Section 2. The problem statement is introduced in Section 3. Section 4 explains the proposed algorithm design in detail. The simulation utilized to evaluate the proposed algorithm is introduced in Section 5, with results discussed in Section 6. The paper concludes in Section 7.

## 2. Related Works

Scalar field based localization system designs have been researched in applications such as gravity-aid navigation [11,18,19], magnetic anomaly–based navigation [7,20], and terrain-based navigation [21]. In order to perform robust localization in featureless areas or with low-quality sensors, cooperative multi-agent localization systems are proposed to achieve accurate estimations [22,23,24]. Distributed multi-agent localization methods were first formulated based on Kalman filters [25,26]. Even though these methods allow to perform an observation update and data exchange when agents are within the communication range, each agent in the group is required to estimate the poses of all agents, which does not scale well to large groups. Meanwhile, the Kalman filter–based estimation methods assume that the pose estimate can be presented by a unimodal Gaussian distribution. However, the scalar field–based estimation error distributions are usually multi-modal and difficult to be approximated by the Gaussian distribution [7,12].

Canciani et al. formulated the magnetic anomaly–based cooperative navigation problem as a particle filter [14]. The method does not scale for large groups, due to the use of a centralized particle filter. Our previous works in [6,15] broke the localization process into two steps: the relative poses between agents are estimated using inter-agent ranging measurements through an extended Kalman filter (EKF), and then each agent estimates its pose using all magnetic anomaly measurements and relative poses of the group through a particle filter. Although the particle filter in [6,15] only contains four states, the EKF formulation, which includes all agents’ poses, does not scale to large group sizes.

A decentralized cooperative bathymetry based localization method was proposed in [17]. In [17], each agent is able to estimate its pose through matching altimeter measurements with a bathymetric map, using a marginalized particle filter. Then, the Gaussian belief, estimated based on the inter-agent ranging measurement and the other agent’s position estimate, is applied to update the particles in the filter. Although this method is able to achieve scalable cooperative localization, it ignores the correlation of the information, which may lead to over convergence. Rui et al. then presented an extended information filter to address the issues about the correlation of the information [27]. However, the method described in [27] is reliable to GPS measurement or highly accurate bathymetric information–based estimations for the prediction update, which leads this method to be non-feasible in an underwater environment.

Wiktor et al. presented a decentralized CI based collaborative multi-agent localization algorithm applied in natural terrain-aid navigation [12]. Similar as [17], each agent is assumed to perform terrain-aid navigation to estimate its own pose and related covariances. The pose estimates are updated using inter-agent ranging measurements and agents’ pose and covariances through a CI filter, which can fuse estimates with unknown correlation. One potential limitation of this method is the robustness within feature-poor regions due to the single measurement used in map matching for each agent at each time step. Compared with those methods presented in [12,17,27], which only use the information from the immediate neighbors, in our proposed algorithm, a subgroup strategy, instead of utilizing single measurements, is applied to improve the robustness of the localization system.

Active multi-agent navigation algorithms [28,29], which combine localization and active path planning algorithm, are interesting research directions to improve the robustness of the pose estimation. However, to the best knowledge of the authors, there is so far no active multi-agent navigation algorithms focused on localization based on map matching using scalar field information.

## 3. Problem Statement and Notations

In this study, the case is considered where a large group of *N* agents (e.g., space, aerial, ground, surface, or underwater vehicles) is entering an environment without GNSS. At the moment entering the GNSS-denied environment, the initial pose in the global frame for each agent is assumed to be known with a small uncertainty (e.g., provided by GNSS). The main objective is to achieve accurate and robust global localization for all agents in a large group mainly based on local communication and inter-agent ranging measurements along with scalar field information. In this paper, a one-based numbering system is used for indexing. Let the group of *N* agents be denoted by $\Phi =\{\phi_{1},\phi_{2},\cdots ,\phi_{N}\}$ where $\phi_{i}$ denotes the *i*th agent in the group. The pose of each agent is partially observable; thus, at each time step, the pose is represented as a Gaussian belief $\phi_{i}\left[b_{k}\right]=\left\{\phi_{i}\left[{\hat{x}}_{k}\right],\phi_{i}\left[P_{k}\right]\right\}$ where $\phi_{i}\left[{\hat{x}}_{k}\right]$ and $\phi_{i}\left[P_{k}\right]$ are the mean vector and covariance matrix of the state of the *i*th agent at the *k*th time step.

Each agent is assumed to be equipped with radios that enable communication with nearby agents to exchange information, as well as to perform undirected inter-agent ranging. Due to communication limitations (e.g., range or number of channels), each agent can only communicate and perform ranging with a limited number of agents inside a certain range. Therefore, the agents are divided into subgroups based on the communication constraints as follows:

(1) $$\begin{array}{c} \Phi_{i}=\left\{\phi_{j}\in \Phi \;|\;\zeta \left(\phi_{i},\phi_{j}\right)<\epsilon \right\} \end{array}$$

where $\zeta (\phi_{i},\phi_{j})$ is a user defined function indicating if agent *i* is capable of communicating with agent *j* and ϵ is a user defined threshold. For example, $\zeta (\phi_{i},\phi_{j})$ may be based on signal strength, and ϵ may define a threshold determining whether the signal strength is adequate for agent *i* to communicate with agent *j*.

Based on this definition, the group has at least *N* subgroups, each with *M* members, where subgroup $\Phi_{i}$ corresponds to the agents capable of communicating with agent $\phi_{i}$. The agent *i* is named the fusion center agent of the subgroup *i*. Furthermore, the subgroups are not disjointed (i.e., $\Phi \neq {⋃}_{i=1}^{N}\Phi_{i}$), as each agent is included in multiple subgroups. In other words, the situations of isolated agents and isolated subgroups are not considered in this study. The beliefs of subgroup *i* at time step *k* are denoted by the following:

(2) $$\begin{array}{c} \Phi_{i}\left[b_{k}\right]=\left\{\phi_{j}\left[b_{k}\right]\;|\;\phi_{j}\in \Phi_{i}\right\} \end{array}$$

where $\Phi_{i}\left[{\hat{x}}_{k}\right]$ and $\Phi_{i}\left[P_{k}\right]$ are similarly the set of means and covariance matrices of subgroup *i* at time step *k*. Each agent in a subgroup measures the distance between other agents in the subgroup. Let the distance between agent *i* and agent *j* be denoted by $d[\phi_{i},\phi_{j}]$, and let the set of distance measurements between agents in a subgroup be denoted by $\Phi_{i}\left[d\right]$.

Each agent is assumed to be loaded with a prior scalar field map covering the operation area. Since the types of scalar fields evaluated in this study change very little in a short time frame [7,8], the prior loaded scalar field map is assumed to be deterministic. Each agent also performs real-time measurements of the scalar field at the current location with sensor noises, such as gravity anomaly, magnetic anomaly, or altimeter measurements, at each time step, which can be exchanged through the communication links. In general, scalar fields may vary with altitude depending on the applications (e.g., magnetic anomaly) and the available data at different altitudes, which may not be available or dense enough; however, this study only deals with the navigation problem in 2D (i.e., assuming the agents are moving at a constant altitude). The scalar field measurements of the *i*th agent are denoted by $\phi_{i}\left[I\right]$, and the sets of these measurements in a subgroup are denoted by $\Phi_{i}\left[I\right]$.

Meanwhile, each agent is assumed to be able to measure or estimate its velocity at each time step, which could be achieved through utilizing the Doppler velocity log [30], wheel odometry [31], or vehicle dynamic model [32], along with yaw rate measurements, using gyroscopes. Similar to before, the velocity and yaw rate measurements of the *i*th agent are denoted by $\phi_{i}\left[v\right]$ and $\phi_{i}\left[\omega \right]$, and the sets of measurements in a subgroup are denoted by $\Phi_{i}\left[v\right]$ and $\Phi_{i}\left[\omega \right]$.

## 4. System Design

### 4.1. System Overview

**Figure 2:**
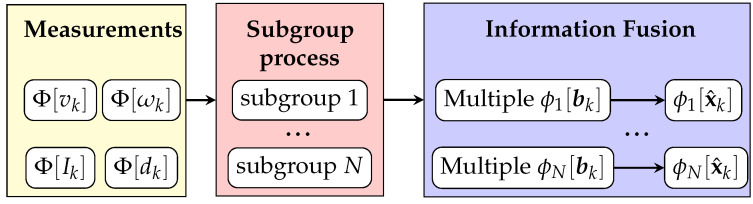
The pipeline of the proposed algorithm. The details of the subgroup localization process are shown in Figure 3.

The proposed algorithm is developed for estimating each agent’s global pose in a scalable group with communication constraints. The pipeline of the proposed multi-subgroup based algorithm is shown in Figure 2.

Each agent in the group is assigned to different subgroups based on the communication range or the number of channels, and these subgroups overlap, as discussed earlier in Section 3. Note that in this study, in order to conveniently evaluate the performance of the proposed algorithm for each agent in the group, the subgroups are pre-set at the beginning, which means that the memberships of each subgroup are assumed to be constant during an operation. Without pre-setting the subgroups, each agent in the group cannot be guaranteed to belong to the same subgroups during the operation, which could lead the performance of each agent to be unequal and difficult to evaluate. Each subgroup takes each agent’s velocity, yaw rate, scalar field measurements, as well as the ranging measurements between the agents in the subgroup as inputs. The outputs of each subgroup are pose estimates of the agents belonging to this subgroup. As mentioned earlier, each agent could belong to multiple subgroups. Thus, multiple pose and covariance estimates from different subgroups exist for each agent in the group at each time step. For example, each subgroup contains *M* agents and each agent is a member of *L* subgroups. Therefore, *L* estimates of the pose and covariance can be communicated to that agent. For each agent, the information fusion algorithm, explained in Section 4.3, is developed for estimating the agent’s pose based on this redundant information.

### 4.2. Subgroup Cooperative Localization

**Figure 3:**
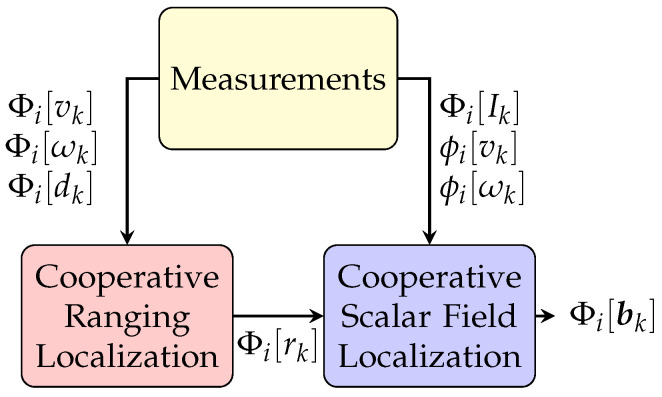
The pipeline of the cooperative localization process in a subgroup *i*. $\Phi_{i}\left[r_{k}\right]$ means the relative poses between each pair of agents in the subgroup *i* at time step *k*.

The cooperative localization algorithm that was developed in our previous work [6] is applied to obtain the estimated poses and covariances of all agents in a subgroup at each time step. For completeness, the main approach and key equations are explained in this subsection. The pipeline of the subgroup process is described in Figure 3. Specifically, two sequential algorithms, named cooperative ranging localization and cooperative scalar field localization, are included in this process and performed in the fusion center agent in each subgroup. The cooperative ranging localization step estimates the relative poses between each pair of agents in the subgroup. The cooperative scalar field localization step then estimates the pose and covariances of the fusion center agent in the subgroup through matching the multiple scalar field measurements with relative pose constraint to the prior scalar field map. Since the processes in the rest of this subsection are identical for each subgroup, in order to present equations clearly, all notations listed in the rest of this subsection do not include the index of the subgroup.

#### 4.2.1. Cooperative Ranging Localization

The cooperative ranging localization algorithm is formulated as an EKF with the state vector given by the following:

(3) $$x={\left[\pi^{\left(1\right)},\;\pi^{\left(2\right)},\;\ldots ,\;\pi^{\left(M\right)}\right]}^{T}$$

where $\pi^{\left(i\right)}={\left[x^{\left(i\right)},\;y^{\left(i\right)},\;\theta^{\left(i\right)}\right]}^{T}$ is the pose of the *i*th agent in the subgroup in the global frame where *x* and *y* are the Cartesian coordinates for the position, θ is the heading, and *M* is the number of agents in the subgroup. The states, shown in Equation (3), at the next time step are predicted using each agent’s velocity and yaw rate measurements and current states. The inter-agent ranging measurements are applied to perform observation updates. The observation function is given by the following:

(4) $$h\left(x_{k|k-1}\right)=\sqrt{{\left(x^{\left(i\right)}-x^{\left(j\right)}\right)}^{2}+{\left(y^{\left(i\right)}-y^{\left(j\right)}\right)}^{2}}:\left(i\neq j\right)$$

where $\left(x^{\left(i\right)},y^{\left(i\right)}\right)$ and $\left(x^{\left(j\right)},y^{\left(j\right)}\right)$ are Cartesian coordinates of an arbitrary pair of agents in the subgroup. At each time step, the relative pose of each agent with respect to other agents in the Cartesian coordinates can be calculated as the following:

(5) $$\left[\begin{array}{c} {\hat{x}}^{\left(i/j\right)}\\ {\hat{y}}^{\left(i/j\right)}\\ {\hat{\theta }}^{\left(i/j\right)} \end{array}\right]=\left[\begin{array}{c} {\hat{x}}^{\left(i\right)}\\ {\hat{y}}^{\left(i\right)}\\ {\hat{\theta }}^{\left(i\right)} \end{array}\right]-\left[\begin{array}{c} {\hat{x}}^{\left(j\right)}\\ {\hat{y}}^{\left(j\right)}\\ {\hat{\theta }}^{\left(j\right)} \end{array}\right]$$

where ${\left[{\hat{x}}^{\left(i/j\right)},{\hat{y}}^{\left(i/j\right)},{\hat{\theta }}^{\left(i/j\right)}\right]}^{T}$ are the relative pose for agent *i* with respect to agent *j* in the subgroup, ${\left[{\hat{x}}^{\left(i\right)},{\hat{y}}^{\left(i\right)},{\hat{\theta }}^{\left(i\right)}\right]}^{T}$ and ${\left[{\hat{x}}^{\left(j\right)},{\hat{y}}^{\left(j\right)},{\hat{\theta }}^{\left(j\right)}\right]}^{T}$ are the pose estimates of agent *i* and agent *j* in the subgroup from EKF, respectively.

#### 4.2.2. Cooperative Scalar Field Localization

With the estimated relative poses in the subgroup, the scalar field measurements from each agent can be treated as points on a fix geometric shape to match to the map. The cooperative scalar field localization method is formulated as a particle filter involving only four states as follows:

(6) $$p={\left[x,y,\theta ,\gamma \right]}^{T}$$

where *x* and *y* are the Cartesian coordinates for the global position of the fusion center agent in the subgroup, θ is the heading of the agent, and γ is the rotation error of the subgroup, which is explained in detail in [15]. In brief, the rotation error is defined as the rotation angle in the global frame of the estimated geometric structure of the subgroup from the cooperative ranging localization. This is necessary since the EKF in the cooperative ranging localization is only capable of preserving the pairwise distances, and as a result, only the geometric structure of the subgroups are maintained through the EKF. The relative position estimates of each agent with respect to the other agents in the subgroup can be corrected based on γ by the following:

(7) $$\left[\begin{array}{c} {\overset{‘}{x}}^{\left(i/j\right)}\\ {\overset{‘}{y}}^{\left(i/j\right)} \end{array}\right]=\left[\begin{array}{cc} \cos\gamma & -\sin\gamma \\ \sin\gamma & \cos\gamma \end{array}\right]\left[\begin{array}{c} {\hat{x}}^{\left(i/j\right)}\\ {\hat{y}}^{\left(i/j\right)} \end{array}\right]$$

where ${\left[{\overset{‘}{x}}^{\left(i/j\right)},{\overset{‘}{y}}^{\left(i/j\right)}\right]}^{T}$ are the relative position coordinates for agent *i* with respect to agent *j* in the subgroup after applying the update utilizing γ, and ${\left[{\hat{x}}^{\left(i/j\right)},\;{\hat{y}}^{\left(i/j\right)}\right]}^{T}$ are the relative position coordinates for agent *i* with respect to agent *j* in the subgroup from Equation (5).

Since the particle filter is performed in the fusion center agent, for each particle, the states, shown in Equation (6), at the next time step are predicted, using the fusion center agent’s velocity and yaw rate measurements and the state estimate at the current time step. Note that since the rotation error changes slightly and is difficult to predict, the state γ is propagated by a random walk. The observation model for each particle is given by the following:

(8) $$y=h_{M}\left(p,\;r\right)+\eta$$

where y is the observation vector with length *m* and η is the measurement noise vector. The relative position r is calculated from Equation (5). Therefore, in the subgroup, the other agents’ predicted global positions can be extracted by adding the updated relative positions from Equation (7) to the predicted states from the particle filter. The vector-valued observation function $h_{M}$ is used to extract the predicted scalar field measurements from the given map based on each agent’s predicted position.

### 4.3. Information Fusion

The goal of the information fusion step is to estimate an improved pose for each agent. Since the correlation among the pose and covariance estimates is unknown due to information reuse between subgroups, the CI algorithm [33] is applied to fuse the estimates of each agent from multiple subgroups. In general, the CI algorithm fuses information based on a convex combination of the information matrices (i.e., the information states and the inverse of corresponding covariance matrices). Therefore, to apply CI, each agent must have knowledge of the expectations and associated covariance matrices estimated in the subgroups it belongs to. The procedure for obtaining these values is described in detailed in this section.

#### 4.3.1. Covariance Estimates from Particles

The expectation of the state at time *k* for the fusion center agent in each subgroup, denoted as ${\hat{p}}_{k}$, can be obtained from the particle filter by computing the weighted average of the particles given the particle weights. Let ${}^{l}p\left(i\right)$ denote *i*th state of the *l*th particle, and let $\hat{p}\left(i\right)$ denote the expected value of the *i*th state. Now, the covariance matrix of the state denoted by *C* is calculated from the particles as follows:

(9) C(l,p)=∑i=1nw˜ipi(l)−p^(l)pi(p)−p^(p)

where ${}^{i}\tilde{w}$ is the weight of the *i*th particle, *n* is the total number of particles, and *l* and *p* represent the indices of the elements in the matrix. Then, the covariance matrix of the pose estimate of each agent at time step *k* is given by the following:

(10) $$\phi_{i}\left[P_{k}\right]\left(l,p\right)=C_{k}\left(l,p\right):\left(l,p=1,2,3\right)$$

where $\phi_{i}\left[P_{k}\right]\left(l,p\right)$ denotes the element in the *l*th row and *p*th column of the covariance matrix of *i*th agent. The pose estimates of other agents in the subgroup are able to be derived by utilizing the fusion center agent’s pose estimate, the relative pose from Equation (5), and the subgroup rotation error γ. According to [34], the covariance matrix of the pose estimate for each agent in the subgroup at one time step is the same as the covariance of the fusion center agent.

#### 4.3.2. Covariance Intersection

In order to present the details of CI applied in this problem clearly, ($\phi_{i}\left[{\hat{x}}_{k}^{j}\right]$, $\phi_{i}\left[P_{k}^{j}\right]$) denotes the *j*th estimate of pose and covariance of *i*th agent at time step *k*. The implementation of CI in this situation is presented as follows:

(11) $$\phi_{i}\left[{\hat{x}}_{k}\right]=\phi_{i}\left[P_{k}\right]\sum_{j=1}^{L}\left(\alpha_{j}{\phi_{i}\left[P_{k}^{j}\right]}^{-1}\phi_{i}\left[{\hat{x}}_{k}^{j}\right]\right)$$

(12) $$\phi_{i}\left[P_{k}\right]={\left[\sum_{j=1}^{L}\left(\alpha_{j}{\phi_{i}\left[P_{k}^{j}\right]}^{-1}\right)\right]}^{-1},\sum_{j=1}^{L}\alpha_{j}=1,\alpha_{j}\geq 0$$

where $\alpha =[\alpha_{1},\alpha_{2},\cdots ]$ are the weighting coefficients, which need to be solved, and *L* is the total number of solutions of the *i*th agent (i.e., the total number of subgroups, including agent *i*). Based on [33], α is assumed to be computed by solving a nonlinear optimization problem, which is to find α to minimize the determinant or trace of $\phi_{i}\left[P_{k}\right]$. However, to solve the nonlinear optimization problem is computationally expensive, especially, when the *i*th agent has a large number of solutions. To reduce the computation cost, the fast CI method presented in [35], which is designed to solve the weighting coefficients quickly, is applied as follows:

(13) $$\alpha_{j}=\frac{\det\left(S\right)-\det\left(S-\phi_{i}\left[P_{k}^{j}\right]\right)+\det\left(\phi_{i}\left[P_{k}^{j}\right]\right)}{L\det\left(S\right)+\sum_{q=1}^{L}\left[\det\left(\phi_{i}\left[P_{k}^{q}\right]\right)-\det\left(S-\phi_{i}\left[P_{k}^{q}\right]\right)\right]}$$

with

(14) $$S=\sum_{q=1}^{L}\phi_{i}\left[P_{k}^{q}\right].$$

By solving for α from Equation (13), the improved pose of the agent at time step *k* (i.e., $\phi_{i}\left[{\hat{x}}_{k}\right]$) can be computed through Equations (11) and (12).

## 5. Simulation

In order to verify that the proposed algorithm is suitable for different types of scalar fields, a magnetic anomaly map and a bathymetric map are utilized in the simulation study.

For the ease of simulating a large number of agents, the agents are simulated with a bicycle model [36] in the 2D plane. A feedback controller steers an agent to follow a reference trajectory and reference velocity based on the pose estimates from the proposed algorithm. All reference trajectories are set to be parallel to latitude lines in a geographic coordinate system. New parallel reference trajectories are added with a certain distance when the group size increases. Agents are assumed to be moving from west to east with the same initial longitude and small uncertainties (a Gaussian white noise with zero mean and 1 m standard deviation) on the initial poses as discussed in Section 3. In order to simulate situations when all agents are moving at different speeds, the reference velocity for each agent varies slowly, following a sine function with a random offset throughout the duration of the mission.

In this simulation, the communication among agents is restricted by the limited number of communication channels available for each agent. The fusion center agent and agents it can reach are assigned as a subgroup at the beginning of the simulation. Meanwhile, different scalar fields are related to different experimental environments and platforms. For example, magnetic anomaly maps can be used by the localization system of aerial vehicles on Earth [7], or space probes exploring other planets, and bathymetric maps are used for underwater vehicles’ localization [12]. Therefore, different parameter settings are introduced corresponding to two map types in the following.

### 5.1. Magnetic Anomaly Map

**Figure 4:**
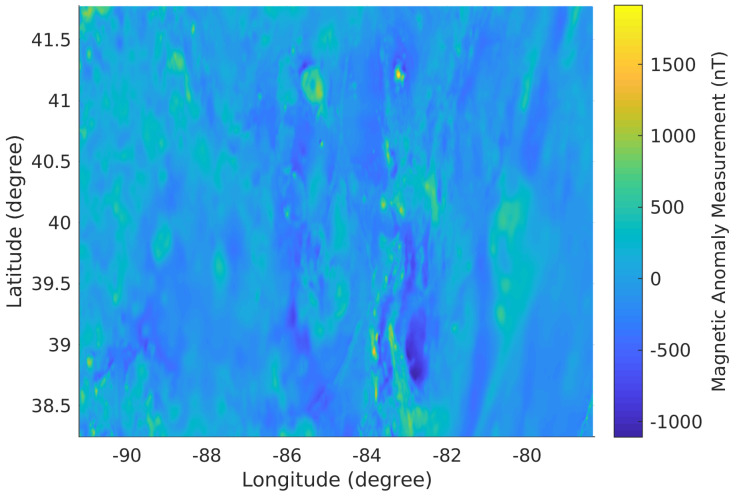
The magnetic anomaly map of the area around Columbus, Ohio, United State in geographic coordinate system at 305 m altitude.

The Earth’s magnetic anomaly information that presents the variations of the Earth’s magnetic field is stable and distinctive at different locations in a certain range of altitude [7]. The magnetic anomaly map utilized in this simulator, shown in Figure 4, is obtained from the United States Geological Survey [37] and contains the magnetic anomaly information at 305 m altitude from the area around Columbus, Ohio, United States.

The noise of the velocity measurement and yaw rate measurement are drawn from a Gaussian distribution with zero mean and standard deviation $\sigma_{v}=0.3$ m/s for velocity and $\sigma_{g}=0.005$ deg/s for the yaw rate. A turn-on bias $b_{v}∼N\left(0,0.1\sigma_{v}\right)$ for velocity and $b_{g}∼N\left(0,0.1\sigma_{g}\right)$ for yaw rate is added separately. According to the references [7,38], the noise of the ranging measurements and the magnetic anomaly measurements are drawn from a Gaussian distribution with zero mean and standard deviation $\sigma_{r}=1$ m and $\sigma_{m}=10$ nT, respectively. The initial distance between each pair of neighbor agents in latitude is set to 1000 m. The reference velocity for each agent varies from 40 to 60 m/s. The mission duration is 1 h, and the trajectory length of each agent is about 180 km.

### 5.2. Bathymetric Map

**Figure 5:**
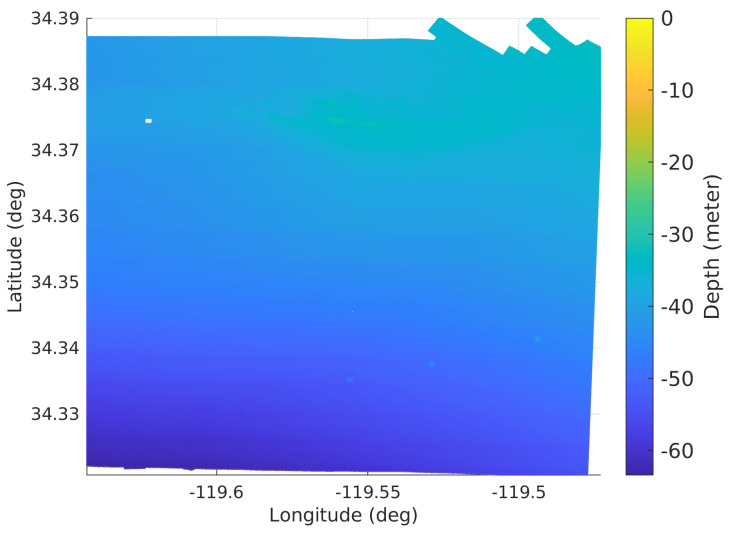
The bathymetric map of the area around the Santa Barbara Channel, United States, in geographic coordinate system.

The bathymetry, also known as submarine topography, presents the depths of the underwater terrain. The bathymetric map applied in this simulator, shown in Figure 5, is acquired from the United States Geological Survey [39].

According to the descriptions of parameter setting in underwater experiments in [12,13], the standard deviation of the velocity measurement noise and yaw rate measurement noise are selected as $\sigma_{v}=0.1$ m/s and $\sigma_{g}=0.1$ deg/s. A turn-on bias $b_{v}∼N\left(0,0.1\sigma_{v}\right)$ for velocity and $b_{g}∼N\left(0,0.1\sigma_{g}\right)$ for the yaw rate is also added separately. The noise of the altimeter measurements is drawn from a Gaussian distribution with zero mean and standard deviation $\sigma_{a}=1$ m. The noise of the inter-agent ranging measurements are drawn from a Gaussian distribution with zero mean and standard deviation $\sigma_{r}=1$ m. The initial distance between each pair of neighboring agents in latitude is set to be 200 m. The reference velocity for each agent varies from 0.5 to 1.5 m/s. The mission duration is 1 h and the trajectory length of each agent is about 3600 m.

## 6. Results and Discussions

The proposed algorithm is designed to remove the constraints of the group size in our previous works in performing cooperative localization [15]. Therefore, the algorithms presented in [15] are utilized as the comparison to evaluate the proposed algorithm. The performance of the proposed algorithm is compared with simulations under the full communication (FC) assumption, where all agents can communicate with each other agent at each time step. The performances and sensitivity analysis of FC could be found in [15]. The average position root mean squared errors (RMSE) in each simulation from both FC and the proposed algorithm are evaluated. In each case, multiple Monte Carlo simulations are performed, consisting of 160 trials each.

**Figure 6:**
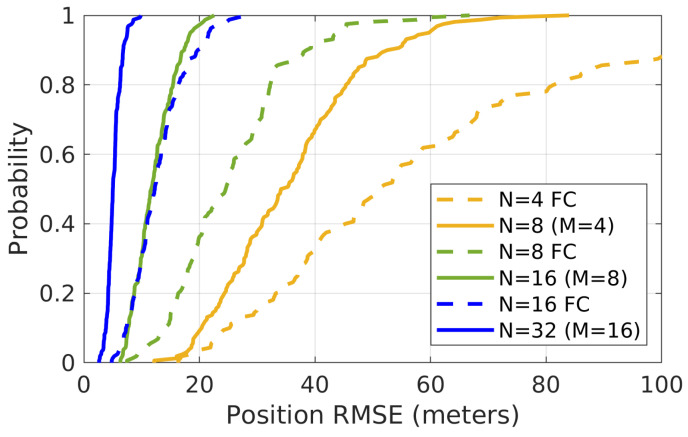
The CDF of each simulation’s average position error for all agents in the group for 160 Monte Carlo simulations with different algorithms and group sizes. These simulations are run with the magnetic anomaly map shown in Figure 4.

The proposed algorithm and FC are first evaluated, using amagnetic anomaly map–based simulation environment. Figure 6 shows the cumulative distribution function (CDF) of each simulation’s average position error for all agents in the group at each time step from the Monte Carlo simulations.

The FC is performed with different group sizes (i.e., N=4,8,16). Meanwhile, the proposed algorithm is also evaluated with various group sizes (i.e., N=8,16,32) and subgroup sizes (i.e., the number of agents in each subgroup), such as M=4,8,16, as shown in Figure 6. For example, N=32,M=16 means that the full group has 32 agents, and each subgroup has 16 agents. Table 1 shows the statistical data of the Figure 6 along with performance of the simulation with dead-reckoning (DR) without using the ranging and magnetic anomaly information.

From Figure 6 and Table 1, it is clear that the performance of both the proposed algorithm and FC improves as the group size increases, and both are much better than doing DR alone. Note that since the number of particles used in different scenarios is the same at the cooperative scalar field localization step, the performance comparison is suitable to be made between FC and the proposed algorithm, when the group size of FC is equal to the subgroup size of the proposed algorithm. In this case, with the same number of particles, the particle filter in both situations deals with the same number of scalar field measurements at each time step. Meanwhile, the computation of each agent applying the proposed algorithm is similar to the computation of each agent working in FC because of the fast CI method [35] applied. The performance of the proposal algorithm shows improvement over the FC in Figure 6 and Table 1.

**Figure 7:**
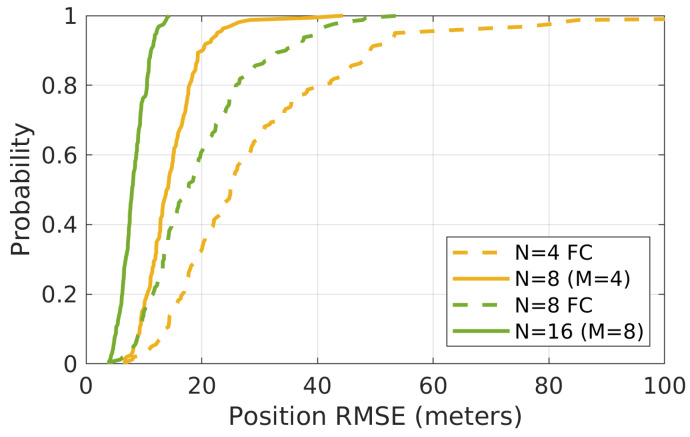
The CDF of each simulation’s average position error for all agents in the group for 160 Monte Carlo simulations with different algorithms and group sizes. These simulations are run with the bathymetric map shown in Figure 5.

Similar results are acquired using a bathymetric map–based simulation environment. Figure 7 shows the CDF of each simulation’s average position error for all agents in the group at each time step. Due to the smaller bathymetric map, a maximum of up to 16 in group sizes were simulated.

Table 2 shows the statistical data of the Figure 7 along with performance of the DR.

The performance of the proposed algorithm applied with different group sizes and the same subgroup size using the magnetic anomaly map is shown in Table 3.

It shows that the proposed algorithm works with large group sizes (e.g., N=128). The computation of each agent in the group, which is only spent on locally centralized cooperative localization and information fusion, does not increase when the group size grows. However, since each agent uses the same amount of information in different sizes of groups (i.e., the sizes of the subgroups are the same), the performance of these simulations are similar.

## 7. Conclusions and Future Work

In this paper, a scalable cooperative localization framework based on scalar field prior maps and real-time measurements is presented. In order to satisfy the communication constraints, a large agent group is separated into several subgroups, where each agent is treated as the fusion center in each subgroup. A locally centralized cooperative localization is performed to estimate the agents’ poses in each subgroup through matching multiple scalar field measurements constrained by relative positions to the given map. In order to avoid over-convergence due to using correlated information, a fast CI algorithm is applied to estimate an improved pose for each agent based on its multiple pose and covariance estimates from its membership in multiple subgroups.

The simulation results show that the proposed algorithm is able to deal with large groups (e.g., N=128), and to achieve higher performance, even under more restrictive communication constraints, compared to our previous works [6,15]. Additionally, this approach is suitable for missions with different types of scalar fields.

There are several limitations in this work that need to be addressed in the future, such as extending the algorithm to agents distributed in 3D spaces and better integration of the uncertainty in the group geometry estimates into the map-matching process. A great challenge is to find decentralized localization solutions that can utilize all agents’ measurements in the group in an efficient and robust manner, under communication constraints. Currently, the performance of the proposed algorithm is limited by the subgroup size, instead of the full group size. Our future work will focus on allowing information to flow beyond the immediate neighbors while maintaining the stability of the pose estimation algorithm in the agent network.

## Figures and Tables

**Table 1:** Mean of Monte Carlo simulations’ position RMSE in each situation, using magnetic anomaly map (unit: meters).

N = 4	N = 8	N = 8	N = 16	N = 16	N = 32	DR
FC	M = 4	FC	M = 8	FC	M = 16	
58.4	35.7	25.3	12.2	12.9	5.2	751.3

**Table 2:** Mean of the position RMSE in each situation using bathymetric map (unit: meters).

N = 4	N = 8	N = 8	N = 16	DR
FC	M = 4	FC	M = 8	
29.6	14.7	19.6	8.2	293.1

**Table 3:** Mean of Monte Carlo simulations’ position RMSE, using magnetic anomaly map with different group size and same subgroup size (unit: meters).

N = 16 (M = 8)	N = 32 (M = 8)	N = 128 (M = 8)
12.2	13.5	15.3

## Data Availability

The code is available online: https://github.com/wvu-irl/Scalable-Framework-Cooperative-Localization.git (Date accessed: 21 September 2021).
